# Early physical activity and clinical outcomes following pediatric sport-related concussion

**Published:** 2020-04-16

**Authors:** Julie C. Wilson, Michael W. Kirkwood, Morgan N. Potter, Pamela E. Wilson, Aaron J. Provance, David R. Howell

**Affiliations:** ^1^Sports Medicine Center, Children’s Hospital Colorado, Aurora, Colorado, United States; ^2^Department of Orthopedics, University of Colorado School of Medicine, Aurora, Colorado, United States; ^3^Department of Pediatrics, University of Colorado School of Medicine, Aurora, Colorado, United States; ^4^Department of Physical Medicine and Rehabilitation, University of Colorado School of Medicine, Aurora, Colorado, United States; ^5^Rehabilitation Medicine, Children’s Hospital Colorado, Aurora, Colorado, United States; ^6^Department of Physical Therapy, University of Delaware, Newark, Delaware, United States

**Keywords:** mild traumatic brain injury, physical activity, recovery, youth

## Abstract

**Objective:**

The objective of the study was to evaluate the clinical outcomes among patients who did and did not report engaging in early physical activity (PA) following sport-related concussion.

**Methods:**

We evaluated pediatric patients seen within 21 days of concussion. The independent variable was early PA engagement (since the injury and before initial clinical evaluation). Dependent variables included demographics, injury details, medical history, Health and Behavior Inventory (HBI) score, and balance, vestibular, and oculomotor function tests.

**Results:**

We examined data from 575 pediatric patients: Sixty-nine (12%) reported engaging in early PA (mean age=14.3±2.4 years; 30% female). The no PA group (mean age=14.5±2.4 years; 35% female) had significantly longer symptom resolution times than the early PA group (median= 16 [interquartile range (IQR)=8-24] vs. 10.5 [IQR=4-17] days; p=0.02). When controlling for pre-existing headache history and time from injury-evaluation time, the early PA group demonstrated lower odds of reporting current headache (adjusted odds ratio=0.14; 95% CI=0.07, 0.26), and reported lower symptom frequency ratings than the no PA group (b=−5.58, 95% CI=−8.94, −2.22).

**Conclusions:**

Patients who did not engage in early PA had longer symptom duration, greater odds of post-injury headache, and greater symptoms at initial clinical evaluation. We cannot determine if patients engaged in early PA due to the lower symptom burden and higher functioning at the time of assessment, or if early PA positively affected outcomes. However, as early PA was associated with better post-injury outcomes, clinicians may consider supervised and structured early PA programs as a method to improve clinical outcomes following concussion.

**Relevance for patients:**

Children and adolescents who were engaged in PA after concussion presented to a clinic with less severe symptoms and had symptoms that resolved sooner compared to those who did not engage in early PA after concussion.

## 1. Introduction

Concussions are a common injury in children and adolescents, with an estimated 1.1-1.9 million sport- and recreation-related concussions occurring every year [1]. Although concussion is generally a self-limited condition, recovery may take longer in children and adolescents compared to adults [2,3]. During this time, concussion symptoms can impact school attendance, sleep patterns, quality of life, and athletic participation [4-8]. To date, few empirically supported treatments are available for concussion [9].

Historically, the cornerstone of concussion management was physical and cognitive rest, which was promoted in the international consensus guidelines on concussion in sport as recently as 2012 [10]. However, more recent expert guidelines now promote a brief (24-48 h) rest period after concussion, followed by gradual reintroduction of physical activity (PA), even if symptoms have not fully resolved [2,3,11]. This evolution in concussion care is based on studies which have found that PA does not negatively impact recovery [12,13], and prolonged or complete rest may actually be harmful [14] among children and adolescents. In addition, regular aerobic exercise below the level of symptom exacerbation appears to be beneficial for symptom reduction among children and adolescents with persistent concussion symptoms [15-18], although the efficacy of exercise on other functional capabilities has not yet been clearly delineated. Further work suggests that it is also safe to perform aerobic exercise in the 1^st^ week after injury [19] and early PA initiation is associated with faster recovery time [20,21], although the dose-response relationship between PA intensity, timing, and frequency, and concussion recovery needs further investigation [22]. However, additional work is needed to clarify whether children or adolescents can begin PA on their own without medical supervision or exercise prescription and still receive similar benefits. In addition, clinical outcomes among children or adolescents who present for their first evaluation to a sports concussion clinic who have already begun exercising are not known. This information would be beneficial for clinicians who see children or adolescents in acute and sub-acute phases of injury to better determine appropriate management and set recovery expectations.

Therefore, the purpose of our investigation was to evaluate if initial post-concussion clinical outcomes vary between children and adolescents (8-18 years of age) who engaged in early PA post-injury relative to those who had not resumed PA when reporting to a sports concussion clinic for initial evaluation within 21 days of injury. We hypothesized that early PA (i.e., beginning PA before the initial evaluation) would be associated with fewer symptoms and better performance on vestibular, ocular, and balance tests.

## 2. Methods

### 2.1. Study design and participants

Patients were seen at the Children’s Hospital Colorado Sports Medicine Center for evaluation of concussion and were enrolled in a prospective clinical registry. We conducted a retrospective investigation of existing clinical records among patients evaluated between January 1, 2015, and August 31, 2017. We included patients who were between the ages of 8 and 18 years, and who were seen for an initial concussion evaluation by a board-certified pediatric sports medicine physician within 21 days of injury. We selected a cutoff of 21 days from injury to initial clinical presentation to examine a sample of participants who were at varying stages of recovery and consistent with the previous studies among children and adolescents with concussion [12,23]. We excluded patients from our analyses who sustained a concussion from a more severe mechanism of injury than typical sport activities (e.g., motor vehicle accident, fall from height; n=229), were over the age of 18 (n=1), were seen more than 21 days from injury (n=73), were not diagnosed with a concussion (n=7), did not answer PA question (n=37), sustained a second head injury before recovery from current concussion (n=4), were not evaluated by a board-certified pediatric sports medicine physician (n=2), had a documented developmental disability (n=2), or had traumatic abnormalities on neuroimaging (n=2). Concussion was defined among the treating physicians consistent with the most recent international consensus guidelines for concussion in sport available at the time of assessment [2,10]. Before study commencement, the local institutional review board approved the protocol.

### 2.2. Grouping variable

We grouped patients dichotomously: Those who reported that they participated in some form of PA since their injury but before the initial evaluation (early PA group), and those who reported that they had not (no PA group). During the evaluation, patients – with assistance from a parent or guardian, if needed – completed an intake questionnaire that included the question, “Are you currently doing any activity/exercise?” If patients reported that they had begun PA or exercise since their injury, they were asked to describe their PA level. Patient responses were clarified by the treating physician during the clinical interview as needed before input into the clinical registry. Responses were categorized into stages corresponding with the graduated return to play protocol, consistent with the previous investigations on PA level following concussion [12,13,20].

### 2.3. Outcome variables

During the clinical examination, patients completed several self-reported questionnaires and clinician-administered tests of balance, vestibular, and oculomotor function. To assess symptomatology, patients and their parents each completed the Health and Behavior Inventory (HBI) symptom frequency questionnaire [24,25]. Patients completed the child version of the HBI by rating the frequency of symptoms experienced on a 4-point Likert scale from 0 (never) to 3 (often) on 20 different concussion symptoms. Total HBI score was then calculated as the sum of responses for all 20 symptoms, where higher scores indicate a higher symptom burden. Parents also completed the parent version of the HBI, which asks the parent to rate their child’s symptom frequency and is scored in the same way as the patient version. Discrepancies between parent and child HBI reports may be clinically relevant, thus, both measures were included in our study [26].

As patients returned for follow-up care through recovery, we also calculated the total symptom duration time, as the time elapsed (days) from injury until the patient no longer reported the presence of any concussion symptoms. Consistent with other studies of child and adolescent patients seen in specialty care concussion clinics [12,27,28], we instructed patients to rate only those symptoms that began at the time of injury and were still present within 1 day of the assessment. Thus, we defined symptom-free as an HBI score of zero. In addition to the HBI, we also assessed the presence of a headache at the time of the initial evaluation. Patients reported their headache severity over the preceding 24 h from 0 (not at all) to 10 (most severe), and we characterized the presence of a headache as any score greater than 0.

Tests of balance, vestibular, and oculomotor function included the Balance Error Scoring System (BESS), Romberg, tandem gait, gaze stability, and near point of convergence tests. Physicians or certified athletic trainers administered all tests, and each of these tests have been described in detail previously [28-35]. The BESS was completed in three different stance conditions (double leg, single leg, and tandem) on firm ground and on a foam pad. The primary outcome measure for the BESS was number of errors committed in each condition. Tandem gait, Romberg, gaze stability, and near point of convergence tests were included as dichotomous variables, recorded as normal or abnormal test performance. A normal tandem gait test was identified by being able to walk without significant loss of balance and to maintain heel/toe contact throughout the test. A normal Romberg test was defined as the patient being able to hold still without significant loss of balance (opening eyes, lifting foot, and falling out of position). An abnormal gaze stability test was identified if patients reported an increase in concussion symptoms (typically dizziness, nausea, headache, or visual problems) during either vertical or horizontal head movements, while a normal test was recorded if they had no increase in concussion symptoms. Finally, a normal near point of convergence test was recorded if the patient reported a diplopia distance of <5 cm [35,36].

To account for additional variables that may have had an effect on the clinical outcome variables, we also assessed several demographic, injury, and pre-injury developmental/medical history variables. These included patient age, sex, and number of days elapsed from injury until the initial concussion evaluation, loss of consciousness (LOC) at the time of injury, and the previous history of diagnosed concussions, Attention-Deficit/Hyperactivity Disorder (ADHD), learning disability, anxiety, depression, and headaches before the concussion. These were documented through standardized questionnaires and obtained before the clinical evaluation.

### 2.4. Statistical analysis

Continuous variables are presented as means (standard deviations) for normally distributed data or medians [interquartile range (IQR)] for non-normally distributed data, and categorical variables are presented as the number included (corresponding percentage). Normality was assessed for continuous variables using Shapiro–Wilk tests. We first compared the demographic, pre-injury history, and injury characteristics between early PA and no PA groups using independent samples *t*-tests, Mann–Whitney U, and Fisher’s exact tests. The characteristics (Table 1) that demonstrated a potential difference between groups (defined as *P*<0.15) were then included as covariates in subsequent multivariable models [37]. These included time from injury to evaluation and history of pre-existing headaches.

**Table 1:** Demographic, medical history, and injury characteristics of the early PA and no PA groups. Continuous variables are presented as medians [interquartile ranges], categorical variables are presented as the number included and percentage of the group.

Variable	Early PA group (n= 69)	No PA group (n= 506)	*P* value
Age (years)	14.7 [13.3-15.8]	14.9 [13.0-16.2]	0.64
Sex (female)	21 (30%)	176 (35%)	0.51
Time from injury to evaluation (days)	12 [8-16]	8 [4-13]	**<0.001^†^**
LOC at time of injury	15 (22%)	84 (17%)	0.31
Prior concussion history	28 (41%)	225 (44%)	0.61
ADHD	10 (15%)	56 (11%)	0.42
Learning disability	6 (9%)	502 (9%)	> 0.99
History of anxiety	2 (3%)	26 (5%)	0.56
History of depression	2 (3%)	9 (2%)	0.63
History of pre-existing headaches	24 (36%)	138 (27%)	0.15†
Sport at the time of injury	Football: 17 (25%) Soccer: 14 (20%) Basketball: 8 (12%) Softball: 4 (6%) Lacrosse: 4 (6%) Skiing: 3 (4%) Cheerleading: 3 (4%) Wrestling: 3 (4%) Skateboard: 3 (4%) Gymnastics: 2 (3%) Unspecified physical activity: 2 (3%) Gym class: 2 (3%) Baseball: 1 (1%) Biking: 1 (1%) Snowboarding: 1 (1%) Rugby: 1 (1%)	Football: 129 (25%) Soccer: 105 (21%) Basketball: 46 (9%) Lacrosse: 35 (7%) Ice hockey: 23 (5%) Volleyball: 20 (4%) Cheerleading: 18 (4%) Unspecified physical activity: 17 (3%) Baseball: 16 (3%) Wrestling: 15 (3%) Snowboarding: 12 (2%) Gymnastics: 8 (2%) Skiing: 8 (2%) Rugby: 8 (2%) Skateboard: 7 (1%) Softball: 6 (1%) Biking: 6 (1%) Equestrian: 6 (1%) Trampoline: 4 (1%) Gym class: 4 (1%) Diving: 3 (1%) Dodgeball: 3 (1%) Swimming: 2 (0%) Ultimate Frisbee: 1 (0%) Tennis: 1 (0%) Water polo: 1 (0%) Martial arts: 1 (0%) Ice skating: 1 (0%)	--

† Variables where *P*<0.15 were included as covariates in subsequent multivariable models

To identify the variables obtained during the initial clinical examination that differed between groups, we first conducted a series of univariable comparisons. We compared continuous outcome variables using Mann–Whitney U-tests, and categorical variables using Fisher’s exact tests. These comparisons were only used for descriptive purposes, no statistical significance inferred.

We then constructed a series of multivariable regression models to identify which outcome variables were associated with early PA. The predictor variable in each model was PA group (early PA vs. no PA) and clinical measures were the outcome variables. We used binary logistic regression models for dichotomous clinical measures (e.g., presence of current headache) and linear regression models for continuous clinical measures (e.g., BESS errors). Statistical significance was set at a<0.05 and all tests were two-sided, and analyses were performed using Stata version 15 (StataCorp, College Station, TX).

## 3. Results

During the study period, we evaluated a total of 934 individual patients for concussion. After applying inclusion and exclusion criteria, we included 575 patients in our analysis. Of these, 69 (12%) reported engaging in PA before their initial clinical examination. The early PA group was seen for their initial post-injury evaluation approximately 4 days later than the no PA group (Table 1). The two groups exhibited similar demographics, injury characteristics, and pre-injury histories (Table 1). Among patients in the early PA group, most indicated participation in light aerobic activity before the initial evaluation, with few reporting participation in more rigorous exercise (Table 2).

**Table 2:** Description of PA stages for the patients who reported engaging in PA between injury and initial evaluation.

PA Stage	Description	N	Days Post-Injury at Initial Evaluation (mean, SD)
1	Light aerobic exercise: Walking or stationary cycling at slow to medium pace	31	11.1 (4.2)
2	Sport-specific exercise: Running or skating drills, no head impact activities	5	14.6 (5.3)
3	Non-contact training drills: Harder training drills, resistance training	8	11.3 (4.2)
4	Full contact practice	0	
5	Normal game play	4	14.8 (2.9)

PA stage information was not available for 21 patients

**Figure 1:**
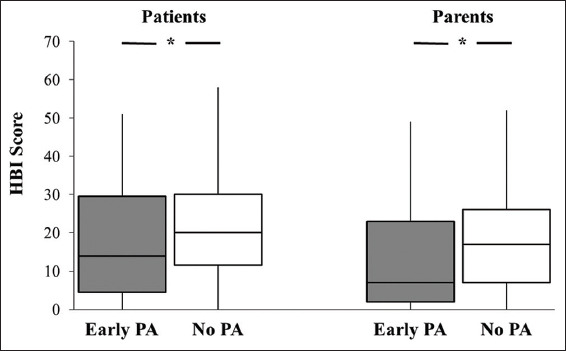
Boxplots of patient-reported and parent-reported symptom frequency as assessed by the Health and Behavior Inventory between patients who did and did not participate in early PA. *For patient-reported symptom frequency, the early PA group demonstrated lower scores than the no PA group (*P*=0.011; Cohen’s d=0.31). *For parent-reported symptom frequency, the early PA group demonstrated lower scores than the no PA group (*P*=0.001; Cohen’s d=0.40).

On univariable analysis, the no PA group had significantly longer median symptom resolution times than the early PA group (16 days [IQR=8, 24] vs. 10.5 days [IQR=4, 17]; *P*=0.02). Patients in the early PA group and their parents also reported significantly lower HBI scores than the no PA group at the initial evaluation (Figure 1). Furthermore, the early PA group consisted of a lower proportion of individuals who experienced headache at the time of initial evaluation and who exhibited an abnormal Romberg test compared to the no PA group (Table 3). In addition, the early PA group committed fewer BESS errors than the no PA group during single leg firm and tandem firm stances (Table 3).

**Table 3:** Comparisons of headache, sleep disturbance, balance, vestibular, and oculomotor function at the initial post-concussion visit between early PA and no PA groups.

Variable	Early PA group	No PA group	*P* value	Odds ratio (95% CI)
Presence of current headache	45 (65%)	464 (92%)	**< 0.001**	0.16 (0.09, 0.29)
Sleep problems since injury	18 (26%)	176 (35%)	0.18	0.66 (0.37, 1.16)
Abnormal tandem gait test	9 (13%)	77 (16%)	0.72	0.83 (0.40, 1.75)
Abnormal Romberg test	7 (10%)	101 (21%)	0.05	0.45 (0.20, 1.02)
Abnormal gaze stability test	25 (37%)	241 (48%)	0.12	0.65 (0.38, 1.09)
Abnormal NPC test	10 (15%)	85 (17%)	0.73	0.85 (0.42, 1.74)

***BESS errors (median, IQR)***	**Early PA group**	**No PA group**	***P* value**	**Effect size (Cohen’s d)**

Double leg firm	0 [0-0]	0 [0-0]	0.36	0.03
Single leg firm	3 [1-5]	4 [2-8]	**0.016**	0.32
Tandem stance firm	1 [0-3]	2 [1-5]	**0.018**	0.29
Double leg foam	0 [0-0]	0 [0-1]	0.44	0.03
Single leg foam	7 [5-10]	8 [6-10]	0.12	0.21
Tandem stance foam	4 [2-6]	5 [3-8]	0.05	0.26

In our multivariable analyses, after adjusting for the independent effect of pre-existing headache history and time from injury until clinical evaluation, early PA was significantly associated with a shorter symptom recovery time (Table 4). Furthermore, patients who engaged in early PA after concussion had significantly lower reports of the presence of a headache during the initial evaluation, lower self- and parent-reported HBI scores, and fewer BESS errors in single leg stance firm, tandem stance firm, and tandem stance foam conditions (Table 4).

**Table 4:** Regression model results for the association between early PA (independent variable) and clinical characteristics (outcome variable) while adjusting for the effect of injury to presentation time and pre-existing headache history.

Dichotomous outcome variables	Adjusted odds ratio	Standard error	95% confidence interval	*P* value
Presence of current headache*	0.14	0.05	0.07, 0.26	**<0.001**
Post-injury sleep problems	0.56	0.17	0.31, 1.00	0.05
Abnormal Romberg test	0.48	0.20	0.22, 1.10	0.08
Abnormal gaze stability test	0.59	0.17	0.34, 1.03	0.06

**Continuous outcome variables**	**β coefficient**	**Standard error**	**95% confidence interval**	***P* value**

Symptom resolution time*	−7.31	2.63	-12.5, -2.11	**0.006**
HBI total score: patient reported*	−5.58	1.71	-8.94, -2.22	**0.001**
HBI total score: Parent reported*	−6.19	1.66	-9.46, -2.92	**< 0.001**
BESS firm ground single-leg stance*	−1.16	0.45	-2.05, -0.27	**0.011**
BESS firm ground tandem-stance*	−1.06	0.42	-1.88, -0.23	**0.012**
BESS foam single-leg stance	−0.64	0.39	-1.39, 0.12	0.10
BESS foam tandem-stance*	−0.95	0.47	-1.88, -0.02	**0.047**

Dichotomous outcome variables were assessed using logistic regression models. Continuous outcome variables were assessed using linear regression models.

* Significantly associated with group (early PA vs. no PA)

## 4. Discussion

In our study, child and adolescent patients who engaged in early PA before the initial evaluation in a sports concussion clinic had shorter symptom recovery times than those who did not. In addition, the early PA group also had lower odds of reporting a headache, better postural stability, and lower patient- and parent-symptom rating at initial evaluation. Given our study design, we cannot determine the causal nature of why patients engaged in early PA (e.g., perhaps due to the lower symptom burden at the time of assessment) or whether early PA actually positively affects clinical characteristics. Our results suggest that there is an association between early PA after concussion and better post-injury outcomes, although causality must be determined through more rigorous prospective study designs.

Our results align with others who found that engaging in early PA after concussion is associated with shorter recovery times [20,21]. However, we also observed that the no PA group was assessed approximately 4 days earlier than the early PA group. After adjusting for this in our multivariable model, we observed that participation in early PA was associated with a symptom duration that was approximately 1 week less than no reported PA. Lawrence *et al*. reported that adolescent and young adult patients seen within 14 days of concussion who initiated aerobic exercise, whether self-initiated or physician-prescribed, had a more favorable recovery including faster return to sport as well as faster return to school or work [20]. Leddy *et al*. recently published a randomized controlled trial demonstrating a median of 4 days shorter recovery time in adolescents completing a sub symptom threshold aerobic exercise program compared to adolescents performing a placebo-like stretching program, initiated within 10 days of sport-related concussion [21].

These past studies focused mainly on self-reported symptom recovery times and return to physical and cognitive activity, but did not address other clinical outcomes for concussion patients. The present study also examined total post-concussive symptom burden and headache severity. A particular strength of our study was that symptom burden was measured not only by self-report but also by parent-report as well. In those reporting early PA engagement, both patients and parents rated total symptomatology at the time of the initial clinic evaluation as significantly less compared to those who did not engage in early PA. In addition, a smaller proportion of the early PA group reported headaches at initial clinical presentation, which may have various explanations. This may be due to factors such as a more severe concussion in the no PA group, although this is a concept that does not have a quantifiable outcome. However, it should be considered as a potential confounding variable, as those who were more symptomatic or affected by the injury were likely limited in their ability to do PA and perhaps independently or as a result, required a longer time to recover. Consistent exercise can reduce symptoms of migraine headaches [38]; therefore, early PA itself may be a factor in improving this symptom. In addition, based on our clinical practice, headaches are a variable that will cause patients to self-limit activity, thus those who had lower headache severity may have been more likely to engage in activity.

Our data indicate that early PA was associated with better postural control outcomes. Participation in early PA may influence postural control through various mechanisms. Cerebrovascular function is associated with better balance control [39]; thus, early PA may have an impact on the vestibular system which could account for improvements in postural tests such as the BESS and Romberg. Recent studies support the use of early vestibular programs to reduce symptom burden and reduce time to return to sport [40,41], but we found no reported associations to date in the literature between vestibular function and early PA. Our study finding, thus, also highlights an area in need of additional research.

Cerebrovascular (or autonomic) dysfunction has also been identified in patients after a concussion [42], and may also underlie dizziness [43,44], which could in turn affect performance on postural control assessments. Transient impairments of the cerebrovascular capillary pressure-buffering system have been reported to persist after 2 weeks in athletes sustaining sport-related concussion which may be due to a prolonged period of autonomic dysregulation. Autonomic dysregulation persisting outside of the acute phase (1-3 days) of recovery may contribute to prolonged dizziness [45]. Sub-symptom threshold aerobic exercise has been found to normalize cerebrovascular dysfunction [46]; thus, participation in early PA may influence the development or persistence of autonomic dysfunction post-concussion.

The clinical implications of our study, as well as other recent research and evidence-based guidelines [2,3,11,20,21], suggest that health-care providers should consider encouraging early PA under well-supervised conditions to facilitate better outcomes sooner after injury, rather than perpetuating the historic convention of rest until symptom-free [10]. To date, the majority of the literature supports physician-supervised, individualized aerobic exercise prescription to facilitate concussion recovery, but additional information regarding the optimal exercise “dosage” (intensity, frequency, and duration) is needed [22]. However, clinicians should administer PA recommendations with careful clinical discretion on a case-by-case basis, as potential harmful effects of unsupervised PA after concussion have not yet been reported. In addition, further investigation into the effectiveness of self-initiated PA compared to individualized exercise prescription is also relevant, especially within the child and adolescent population, where patients may be more likely to engage in free play during recovery.

### 4.1. Limitations

As with any study, ours has limitations. Our clinical registry does not collect the date of initiation of PA, nor the corresponding symptom rating at that time. Thus, given our study design, we are unable to address causality of whether early PA promoted faster recovery and improved clinical outcomes in patients with sport-related concussion, or whether patients engaged in early PA because they were recovering faster. The early PA group was seen for initial evaluation a mean of 4 days later than the no PA group so the additional time elapsed since injury may also have contributed to the lower symptom rating and better post-injury outcomes, although we attempted to mitigate this to the extent possible in our multivariable analyses. Furthermore, PA grouping was based on self-reported data, and not validated with activity or heart rate monitors, so it is possible that patients may have under- or over-reported their PA level. In addition, we did not have inter-rater reliability data for our assessments. Given the small between-group differences for the BESS outcomes, the clinical significance is likely low. Finally, we did not have the ability to analyze what health-care visits may have occurred before presentation to sport medicine concussion clinic; thus, we cannot know if patients decided to begin PA on their own, or if this was advised by another health-care provider. Future methodologically stronger research is needed to definitively determine the causal role of exercise in outcomes after child or adolescent concussion.

## 5. Conclusions

Within the first 3 weeks of sport-related concussion, participation in early PA is associated with shorter symptom recovery times, fewer overall symptoms, and better postural control in child and adolescent patients. Clinicians should consider structured early PA programs as a method to improve clinical outcomes following concussion. In children and adolescent populations, however, closer supervision may be required to avoid potential harmful effects from overexertion. Further investigations into proper exercise dosage during concussion recovery are needed.

### Disclosures

**Financial disclosure statement:** Dr. Howell receives research support not related to this study from the Eunice Kennedy Shriver National Institute of Child Health and Human Development (R03HD094560), the National Institute of Neurological Disorders And Stroke (R01NS100952, R41NS103698, R43NS108823), and MINDSOURCE Colorado Brian Injury Network. The remaining authors have nothing to disclose.

### Conflicts of Interest

The authors declare that they have no conflicts of interest related to the study.
